# Neuroendocrine Tumors of the Gallbladder: A Multicenter Case Series and Systematic Literature Review Indicating Predominantly Non-Aggressive Tumor Behavior and a Common Association with Cholesterol Polyps and Cholesterolosis

**DOI:** 10.1007/s12022-026-09921-3

**Published:** 2026-06-11

**Authors:** Alessandro Vanoli, Yue Xue, Atsuko Kasajima, Rebecca Ruffoni, Zeynep Çağla Tarcan, Nuray Tezcan, Xiuli Liu, Pooja Navale, Ahmed Mohamed, Irene Gullo, Roberto Silva, Giulia Vocino Trucco, Marco Volante, Maria Giulia Disanto, Cecilia Taverna, Elif Kuzucular, Claudio Luchini, Juan Carlos Roa, Irene Esposito, Stefano La Rosa, Burcin Pehlivanoglu, Michelle Reid, Günter Klöppel, Olca Basturk, Volkan Adsay

**Affiliations:** 1 Department of Molecular Medicine, Unit of Anatomic Pathology, University of Pavia, Via Carlo Forlanini 16, Pavia, 27100 Italy; 2 Unit of Anatomic Pathology, Fondazione IRCCS Policlinico San Matteo, Pavia, Italy; 3 Department of Pathology, Northwestern University, Chicago, IL USA; 4 Department of Pathology, TUM School of Medicine and Health, Technical University of Munich, Munich, Germany; 5 University of Pavia, Pavia, Italy; 6 Department of Pathology, Memorial Sloan Kettering Cancer Center, New York, NY USA; 7 Department of Pathology, School of Medicine, Koc University, Davutpaşa Caddesi No: 4, Topkapi, Istanbul, 34010 Turkey; 8 Department of Pathology and Immunology, Washington University in St. Louis, St. Louis, MO USA; 9 Department of Pathology, Unidade Local de Saúde São João, Porto, Portugal; 10 Department of Pathology, Faculty of Medicine of the University of Porto (FMUP), Porto, Portugal; 11 RISE-Health, Faculty of Medicine of the University of Porto (FMUP, Porto, Portugal; 12 Pathology Unit, AOU San Luigi Gonzaga, Orbassano, Turin, Italy; 13 Department of Oncology, University of Turin, San Luigi Hospital, Regione Gonzole 10, Orbassano, Turin, 10043 Italy; 14 Unit of Surgical Pathology, Santa Chiara Hospital, ASUIT, Trento, Italy; 15 Department of Health Science, University of Eastern Piedmont Amedeo Avogadro, Novara, Italy; 16 Surgical Pathology Unit, Azienda Sanitaria Locale, San Giacomo Hospital, Novi Ligure, Italy; 17 Department of Pathology, University of Health Sciences Prof. Dr. Cemil Taşcıoğlu City Hospital, Istanbul, Turkey; 18 Department of Diagnostics and Public Health, Section of Pathology and ARC-Net Research Center, University of Verona, Verona, Italy; 19 Department of Pathology, School of Medicine, Pontificia Universidad Católica de Chile, Santiago, Chile; 20 Institute of Pathology, Medical Faculty, Heinrich-Heine-University Dusseldorf, Dusseldorf, Germany; 21 Unit of Pathology, Department of Medicine and Technological Innovation, University of Insubria, Varese, Italy; 22 Hereditary Cancer Research Center, Department of Medicine and Technological Innovation, University of Insubria, Varese, Italy; 23 Unit of Pathology, ASST dei Sette Laghi, Varese, Italy; 24 Department of Pathology, Dokuz Eylül University Faculty of Medicine, Izmir, Turkey; 25 Department of Pathology, Emory University School of Medicine, Atlanta, GA USA; 26 Present Address: University Hospitals Cleveland Medical Center, Case Western Reserve University, Cleveland, OH USA; 27 Present Address: University of Padua, Padua, Italy; 28 Present Address: Cooper University Health Care, Camden, NJ USA

**Keywords:** Cystic duct, Inflammation, Gall bladder, Neuroendocrine neoplasm, Prognosis

## Abstract

Current data on neuroendocrine tumors (NETs) of the gallbladder and cystic duct (GB-NETs) are highly limited, and the available evidence, largely derived from cancer registry data, suggests that these tumors exhibit a substantially more aggressive clinical behavior than NETs arising at other anatomical sites. We analyzed 26 GB-NETs. Female-to-male ratio: 1.9:1; median age: 50 years. They were typically incidental small (median: 0.8 cm, range: 0.08–2.3 cm) tumors, with 81% classified as grade 1 (G1), either pT1 or pT2, and 38% showing a paraganglioma-like pattern at least focally. Body/fundus tumors were mostly polypoid while neck/cystic duct examples showed mural growth. Symplastic pleomorphism was found in 11%, pseudoglandular structures in 50% and psammomatous intraluminal calcifications in 15%. Pancreatic polypeptide, somatostatin, and gastrin were detected in 5/7 (71%), 5/8 (62.5%), and 4/7 (57%) tested cases, respectively. ISLET1 and ARX were diffusely expressed in all 5 tested cases. Cholesterolosis was common, and six appeared to arise within cholesterol polyps. None had any evidence of metastasis and all 12 patients with available follow-up were alive without disease after a median follow-up of 30 months. Critical review of the literature (79 *bona-fide* GB-NETs) showed that tumors > 2 cm and grade 3 (G3) were enriched among metastatic GB-NETs. In conclusion, GB-NETs are generally indolent neoplasms, in contrast to gallbladder neuroendocrine carcinomas. Tumor size and grade may help predict metastatic potential, while the relatively frequent paraganglioma-like morphology, often associated with diffuse somatostatin expression, can represent a diagnostic pitfall. Their frequent association with cholesterol polyps also suggests a possible etiopathogenetic link.

**Supplementary Information:**

The online version contains supplementary material available at 10.1007/s12022-026-09921-3.

## Introduction

Gallbladder neuroendocrine neoplasms (GB-NENs) are rare and account for approximately 2% of gallbladder malignancies and only 0.2% of all NENs [1–4]. Recent studies utilizing the criteria of the 2019 World Health Organization (WHO) Classification of Digestive System Tumors [1] revealed that neuroendocrine carcinomas (NECs) of the gallbladder (GB-NECs) are very similar to their counterparts in the remainder of the digestive system. They behave very aggressively, and they are often discovered together with an adenocarcinoma component and with glandular precursor lesions [5]. Neuroendocrine tumors (NETs) of the gallbladder and cystic duct (GB-NETs), on the other hand, are reported in the current literature to be more aggressive than NETs in other organs. According to national cancer registries and large databases, the 5-year survival rate ranges between 37 and 44% [6, 7]. The Surveillance, Epidemiology, and End Results (SEER) database reports a median survival of as low as 10 months [6] and the National Cancer Database (NCDB) records metastasis in 40% [7]. Finally, the 5th and 6th editions of the WHO classification of Digestive System Tumors refers to a 10-year survival rate of 36% compared to 80% for bile duct NETs [1, 2]. All of these data have led to the consideration of treating patients with GB-NETs with chemotherapy.

In an attempt to provide clarification to the behavior of GB-NETs and to further investigate the nature of these tumors, we collected a multinational case series of 26 GB-NETs and performed a clinico-pathologic and, where possible, immunohistochemical analysis, along with a critical review of the literature.

## Materials and Methods

### Case Selection

The study was approved by the Institutional Review Board and conducted in accordance with the Declaration of Helsinki.

GB-NETs diagnosed between 2006 and 2025 were retrieved from the pathology archives of participating institutions. Case inclusion required histopathologic confirmation of a well-differentiated NET of the gallbladder or cystic duct, defined according to the *WHO Classification of Tumours of the Digestive System* [2] and the *WHO Classification of Endocrine and Neuroendocrine Tumours* [8]. Tumors with poorly differentiated morphology, those meeting criteria for mixed neuroendocrine–non-neuroendocrine neoplasms (MiNENs), biliopancreatic NETs of uncertain primary site, or metastases from other organs were carefully excluded. One previously reported case [9] was re-evaluated to incorporate additional data.

Clinical information, including patient demographics, presenting symptoms, presence of a hereditary tumor syndrome, tumor location (cystic duct/gallbladder neck vs. body/fundus, based on the macroscopic description provided in the pathology reports), presence of cholelithiasis, endocrine functionality, lymph node and/or distant metastasis status, and follow-up data, was obtained from electronic medical records and pathology reports.

### Histopathologic Evaluation

All available hematoxylin and eosin (H&E)–stained slides were independently reviewed by two pathologists (A.V. and V.A.) with interest in neuroendocrine neoplasia, and any diagnostic discrepancies were resolved by consensus. Tumors were classified and graded according to the current WHO Classification [2]. Given the absence of a dedicated staging system for GB-NETs, the *9th* edition *Union for International Cancer Control (UICC)* staging system for gallbladder carcinoma was applied [10].

Morphologic parameters assessed included tumor macroscopy (polypoid or flat/mural nodular), tumor size, WHO grade, pathologic T stage, necrosis, lymphatic and/or vascular invasion, perineural invasion, and histologic growth pattern. Grade 2 (G2) NETs were further subclassified into G2a (Ki-67 < 10%) and G2b (Ki-67 10%-20%), as this distinction identifies behaviorally distinct subsets in pancreatic NETs (Pan-NETs) [11, 12]. The classic histological patterns (well-defined nested, trabecular, or solid) were distinguished from *nonconventional* patterns (e.g., the paraganglioma-like pattern characterized by Zellballen-like nests separated by delicate fibrovascular septa), described as *“morphologic variants”* in previous studies on Pan-NETs [13, 14]. The invasiveness grade according to criteria recently proposed for Pan-NETs was also recorded [15, 16].

All the cases included in this study displayed nodule formation by the coalescence of nests composed of monomorphic neuroendocrine cells, even though some were small. Small, non-confluent neuroendocrine cell clusters described as “micronests”, “hyperplasia”, or “proliferations” [17] were recorded but excluded from the analysis.

The background gallbladder mucosa was evaluated for coexisting non-neuroendocrine lesions, such as cholesterolosis, cholesterol polyps, adenomyomas, gastric and/or intestinal metaplasia, flat dysplasia, intracholecystic neoplasms, and adenocarcinoma.

### Immunohistochemistry

Immunohistochemical (IHC) stains were performed on formalin-fixed, paraffin-embedded tissue sections. The IHC panel included pan-cytokeratins (AE1/AE3), neuroendocrine markers (synaptophysin and chromogranin A), the proliferation marker Ki-67, somatostatin receptor type 2 A (SSTR2A), hormones (somatostatin, pancreatic polypeptide (PP), gastrin, serotonin, glucagon, and insulin), and transcription factors (ISLET1, ARX, CDX2, SATB2, TTF1, and GATA3). All immunostains (including Ki-67) were performed and assessed in the diagnostic laboratories of the respective institutions, and no centralized review of immunohistochemical slides was performed; therefore, the technical protocols for immunohistochemical staining (including specific antibody clones and platforms) varied across institutions according to local laboratory protocols and over time.

Additional immunostains could not be performed in many cases due to lack of accessibility to the blocks as many cases were received as second opinion consultations, or due to insufficient tissue in some cases, particularly those that were very small.

Tumor cell immunostaining was recorded as either negative or positive. Positive cases were further categorized as diffuse or focal. SSTR2A membranous expression was assessed according to established scoring criteria [18], while the Ki-67 index was evaluated using the hot-spot method in accordance with WHO criteria.

## Systematic Literature Review

A systematic literature review of published GB-NETs was conducted in accordance with PRISMA guidelines, using the PubMed and EMBASE databases. The search strategy is shown in Supplementary Fig. 1 (Supplementary Material 1) and included all records from database inception through April 15, 2025. Non-English publications and non-human studies were excluded. Search results were managed using the Rayyan web-based platform, which was accessible to the senior researcher (A.V.), junior researcher (R.R.), and librarian. The junior researcher independently screened titles, abstracts, and full texts, with any discrepancies resolved by consensus with the senior researcher.

Eligible studies included case reports or case series describing primary well-differentiated neuroendocrine neoplasms of epithelial lineage arising in the gallbladder or cystic duct, as defined by current WHO classifications. Reports using historical terminology (“carcinoid tumor”) were included if the described features met diagnostic criteria for well-differentiated NETs. Ambiguous cases (for example, NENs with a high Ki-67 index or with descriptions that did not clearly indicate well-differentiated histology), as well as poorly documented cases, were jointly reviewed to reach consensus regarding eligibility. Cases in which the consensus was not reached were excluded.

Relevant data were extracted into a Microsoft Excel database and included bibliographic information (author, year), patient demographics (sex, age), clinical data (tumor functionality, hereditary syndromes, cholelithiasis, metastatic status at diagnosis, and outcome), and pathologic parameters (tumor site, size, grade, Ki-67 index, lymphatic, vascular and/or perineural invasion, and immunohistochemical profile).

### Statistical Analysis

Continuous variables were summarized as median and range, and categorical variables as counts and percentages. All statistical analyses were performed using Stata software (release 18.5; StataCorp, College Station, TX, USA). Comparative analyses of patient and tumor characteristics were conducted to evaluate potential differences among GB-NETs based on tumor site (cystic duct/neck vs. body/fundus) and metastatic status at diagnosis. Moreover, the findings of the GB-NET multinational cohort were compared with those of 31 GB-NECs (including 22 MiNENs with a NEC component), that were identified in some of the authors’ institutions and consultation practice and which were the subject of a separate study [5]. Fisher’s exact or Chi-square test was used for categorical variables, and the Mann-Whitney U test for continuous variables. A two-tailed *p*-value < 0.05 was considered statistically significant.

## Results

### Demographic and Clinical Features of the Multinational GB-NET Cohort

Twenty-six cases of GB-NETs met the inclusion criteria [1, 2] as described above. Their clinico-pathologic features are summarized in Table 1. The cohort showed a female predominance (female-to-male ratio, 1.9:1) with a median age of 50 years (range, 23–88 years). One patient had multiple endocrine neoplasia type 1 (MEN1), while all other cases did not appear to have any genetic tumor syndrome (regarded as sporadic). All tumors were nonfunctional, and cholelithiasis was specifically recorded in 38% of patients. None had any clinical evidence of metastasis at the time of diagnosis. Survival information was available for 12 patients, all of whom were alive and disease-free at a median follow-up of 30 months (range, 6–201 months).

**Table 1:** Clinicopathologic Features of the Multinational Cohort of 26 Gallbladder Neuroendocrine Tumors (GB-NETs)

Female/male ratio	17/9 (1.9:1)
Median patient age (range)	50 years (23–88)
Hereditary tumor syndrome, n (%)	1 (4)
Functional tumor, n (%)	0
Tumor site	
Neck/cystic duct, n (%)	13 (50)
Corpus/fundus, n (%)	12 (46)
Unknown, n (%)	1 (4)
Gross pattern, n (%)	
Polypoid growth, n (%)	14 (54)
Nodular/mural growth, n (%)	12 (46)
Tumor size, median (range)	0.8 cm (0.08–2.3)
WHO tumor grade	
G1, n (%)	21 (81)
G2, n (%)	5 (19)
Ki67 proliferative index, median (range)	1% (0.5-8)
pT stage*	
pT1, n (%)	11 (42)
pT2, n (%)	15 (58)
pT3/pT4, n (%)	0
pN stage*	
pN0, n (%)	8 (31)
pN1, n (%)	0
No lymph node submitted, n (%)	18 (69)
Distant metastasis, n (%)^	0
Tumor-related death, n (%)^	0
Cholelithiasis, n (%)	10 (38)
Other gallbladder pathologic alterations	
Cholesterolosis, n (%)	10 (38)
Cholesterol polyp, n (%)	7 (27)
Adenomyoma, n (%)	2 (8)
Gastric and/or intestinal metaplasia, n (%)	8 (31)
Low-grade dysplasia, n (%)	2 (8)
ICPN with associated adenocarcinoma, n (%)	1 (4)
Hyperplasia-like neuroendocrine proliferations, n (%)	1 (4)

*WHO* World Health Organization, *ICPN* intracholecystic papillary-tubular neoplasm

*according the UICC staging system (9th ed.) of gallbladder carcinoma

^information available for only 13 patients

## Pathologic Features of The Multinational GB-NET Cohort

**Fig. 1:**
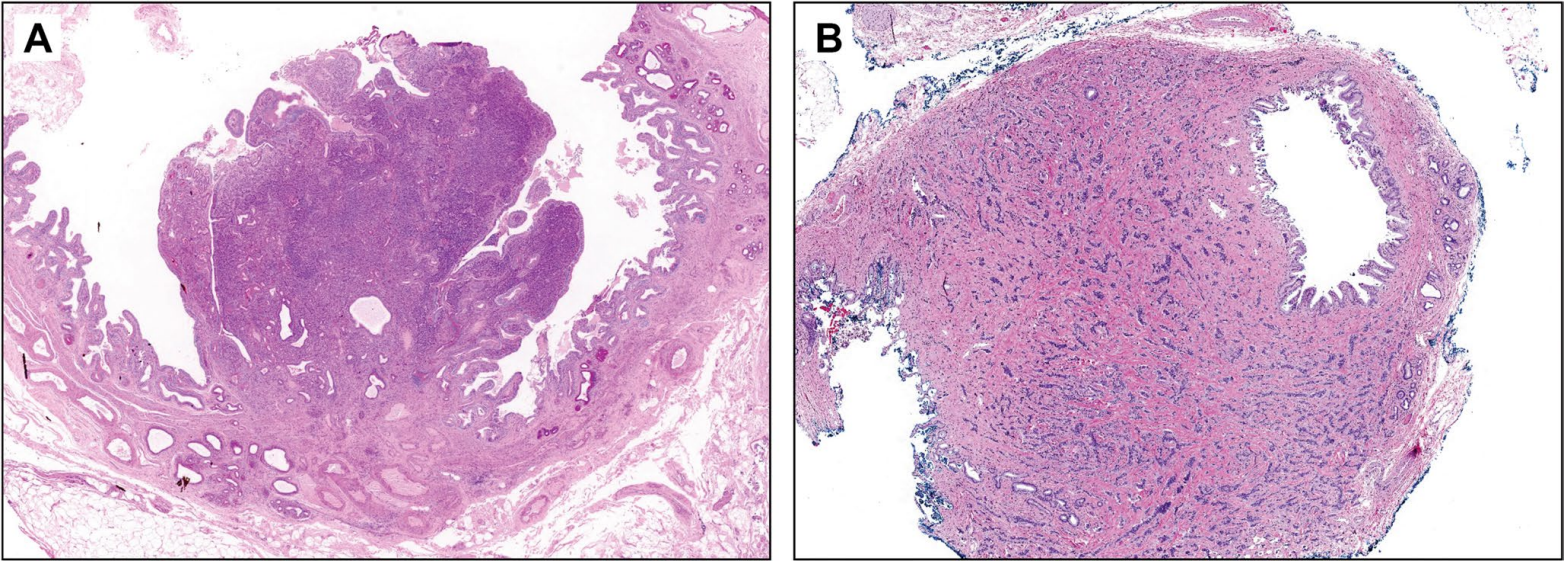
Gross Morphologic Patterns of Gallbladder Neuroendocrine Tumors (GB-NETs): (**A**) Polypoid growth; (**B**) Mural nodular growth

Thirteen tumors (50%) arose in the gallbladder neck/cystic duct, 12 (48%) in the body/fundus, and 1 case had indeterminate site. Grossly, 14 tumors (54%) were polypoid and 12 (46%) were flat or mural nodular (Fig. 1). Median tumor size was 0.8 cm (range, 0.08–2.3 cm). In 9 cases, the tumor was ≥ 10 mm. Twenty-one tumors (81%) were WHO grade 1 (G1) and 5 were G2, each with a Ki-67 index below 10% (range, 3.3–8%); i.e., no G2b or G3 tumors were identified. Mitoses were absent or rare, and necrosis was not seen. In 1 tumor (4%) lymphatic invasion was suspected. Entrapped nerves in the main tumor areas were present in 8 tumors (31%) (Supplementary Fig. 2A), but no extratumoral lymphatic, vascular, or perineural invasion was identified. Eleven tumors (42%) were confined to mucosa and/or tunica muscularis (pT1 level by UICC used for gallbladder carcinomas), with 15 cases (58%) superficially involving the perimuscular soft tissues (pT2 level); none were pT3 or pT4. Lymph nodes were available in 8 cases, all negative for metastasis.

**Fig. 2:**
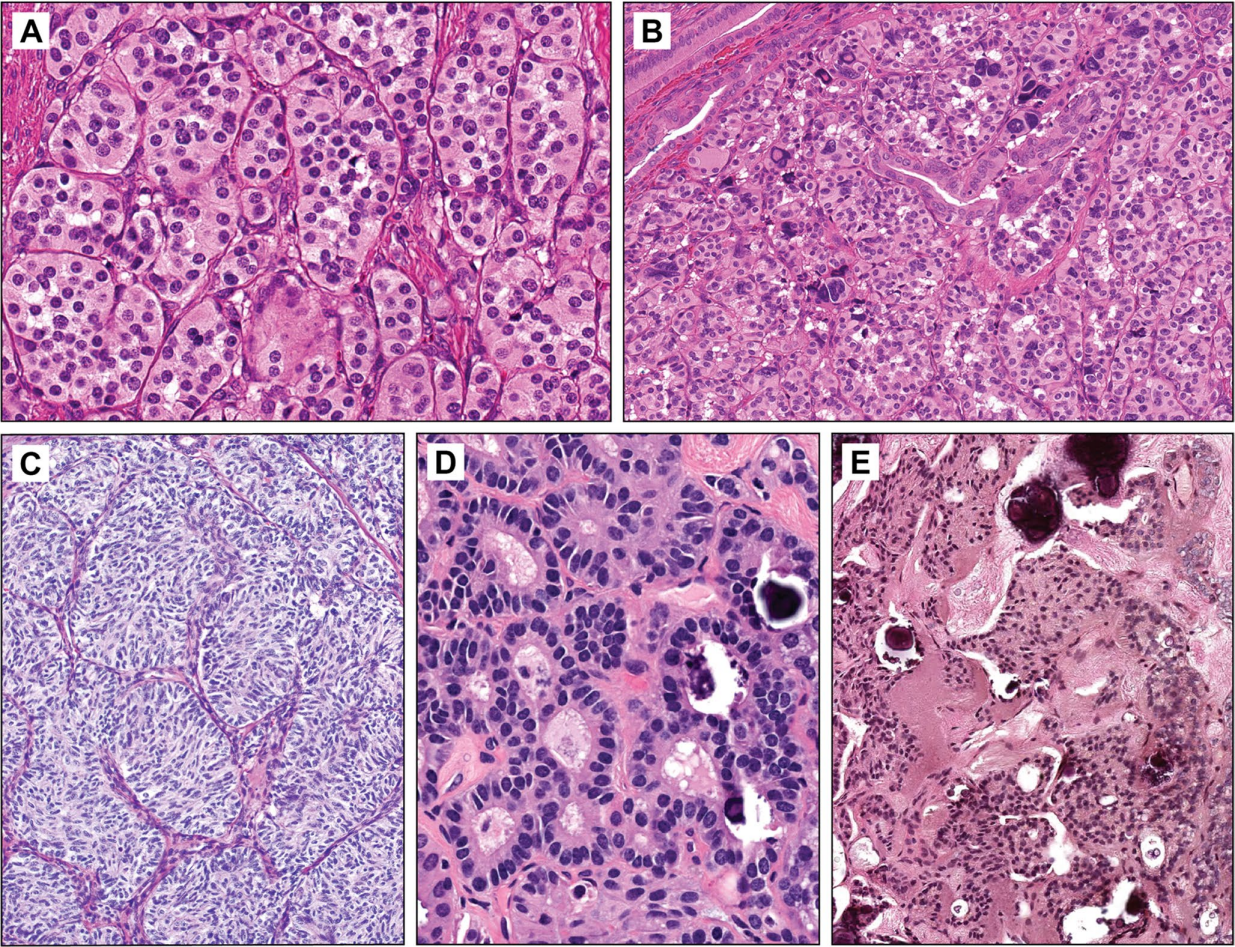
Morphologic spectrum of gallbladder well-differentiated neuroendocrine tumors (GB-NETs). (**A**) Paraganglioma-like pattern (H&E, magnification 100×); (**B**) paraganglioma-like pattern with symplastic/pleomorphic cells (H&E, magnification 100×); (**C**) Spindle cell pattern (H&E, magnification 100x); (**D**) Pseudoglandular pattern with psammoma bodies (H&E, magnification 100x) ; (**E**) Stromal calcification and hyalinization (H&E, magnification 100×)

Microscopically, GB-NETs were well-demarcated, showing noninfiltrative/destructive or only minimal infiltrative growth [15, 16]. Most displayed conventional solid-nested and/or trabecular morphology characteristic of ordinary NETs, although morphologic variants were common. Patterns associated with more benevolent behavior in Pan-NETs included the paraganglioma-like pattern, that occurred in 10 cases (38%; diffuse in 4, focal in 6) (Fig. 2A) and was also accompanied by symplastic/pleomorphic changes in 3 tumors (Fig. 2B), as often seen in Pan-NETs [13]. None of the cases showed oncocytic/hepatoid or more diffuse growth patterns that have been found to be signs of aggressive behavior in Pan-NETs [13]. Focal spindle cell morphology was seen in 4 cases (Fig. 2C) and clear cell changes were noted in 4 (diffuse in 2, focal in 2). A focal pseudoglandular pattern was present in 13 cases (50%) (Fig. 2D), not to be confused with the entrapment of surface mucosa as glandular elements. Psammomatous intraluminal calcifications (Fig. 2D), stromal calcifications, and luminal secretions were observed in 4, 3, and 4 tumors, respectively. Prominent stromal sclero-hyalinization was noted in 6 tumors (Fig. 2E). Entrapped or intermingled non-neuroendocrine glandular elements were observed in 54% of cases.

**Fig. 3:**
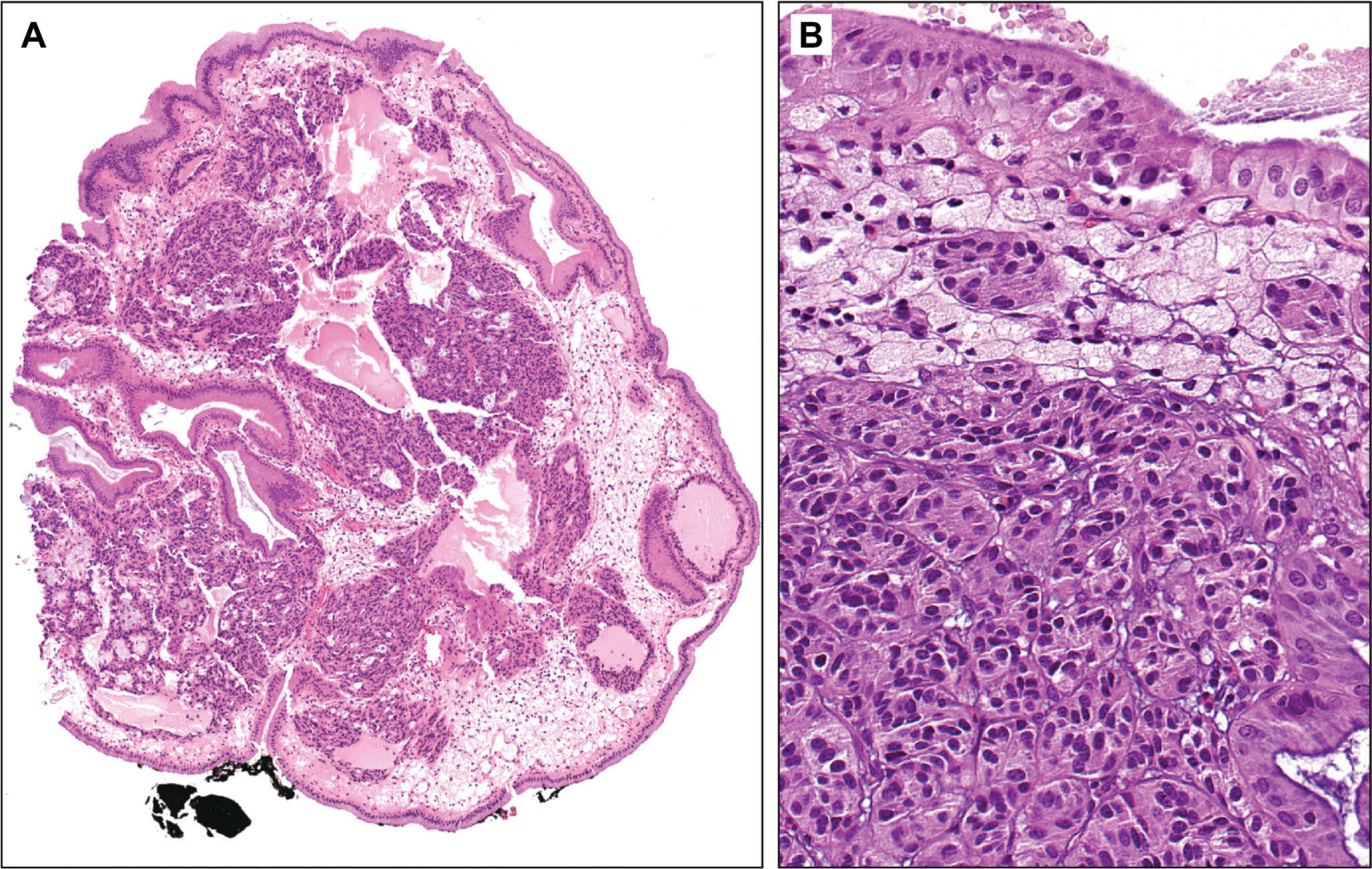
GB-NETs associated with cholesterol polyps or cholesterosis. (**A**) GB-NET arising with a cholesterol polyp (H&E, magnification 40×); **B**. Lipid-laden macrophages (H&E, magnification 100x]

Seven tumors (27%; 4 males and 3 females) were overtly localized within a cholesterol polyp, displaying the typical cauliflower architecture characteristic of cholesterol polyps [19] (Fig. 3). Cholesterolosis was present in 38% of cases and included all cases with cholesterol polyps. Intestinal metaplasia was identified in 8 cases (31%), pseudopyloric metaplasia in 1 case (4%), and low-grade dysplasia in 2 cases (8%). Pancreatic heterotopia was not observed. A hyperplasia-like neuroendocrine proliferation was present in one likely sporadic case. One GB-NET was adjacent to an intracholecystic papillary-tubular neoplasm (ICPN) with associated adenocarcinoma, but without any obvious collision. Two tumors occurred in gallbladders containing isolated adenomyomas, not related to GB-NETs.

### Immunohistochemical Features of the Multinational GB-NET Cohort

**Fig. 4:**
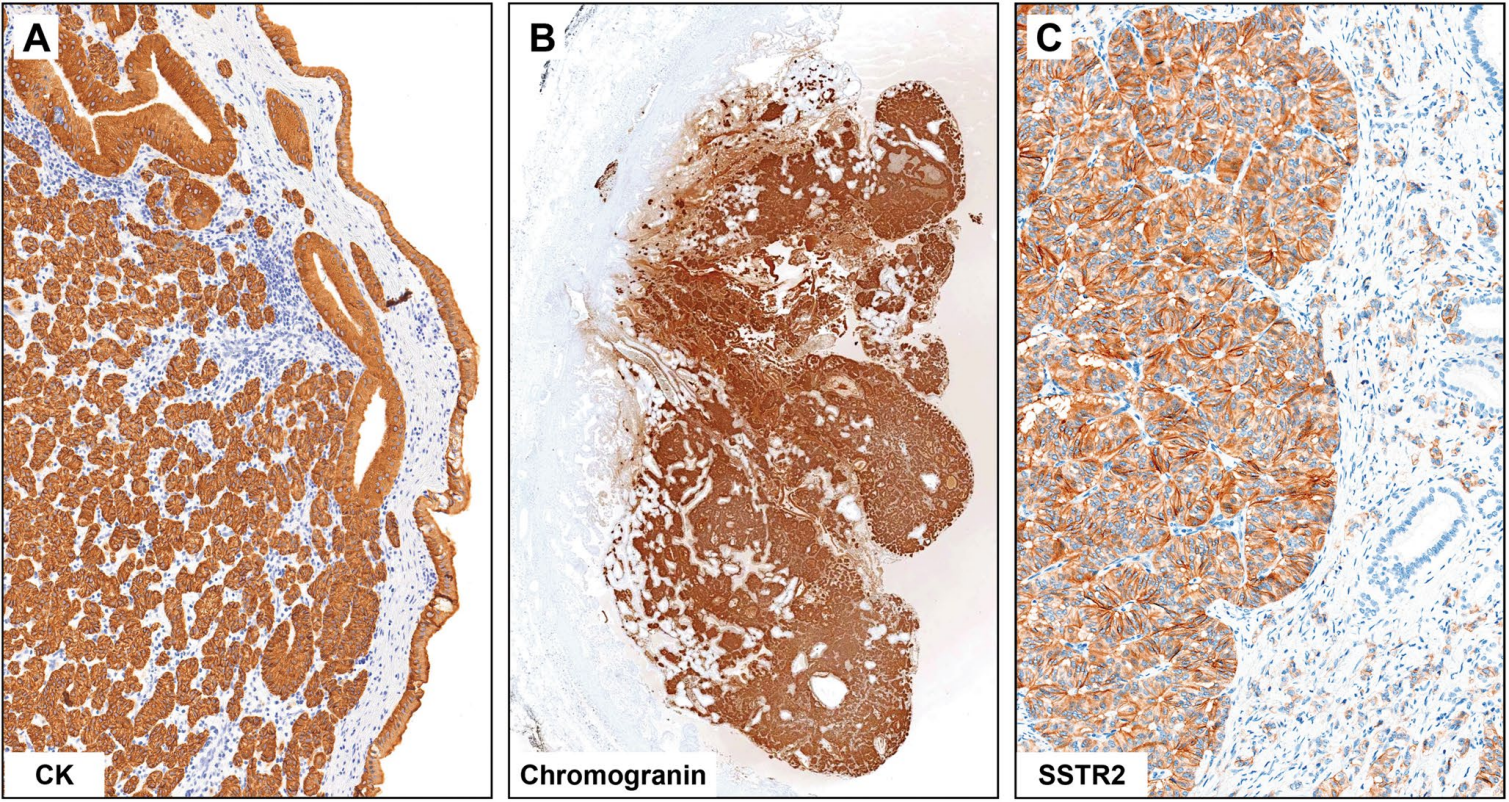
Immunohistochemical features of a gallbladder neuroendocrine tumor (GB-NET): (**A**–**B**) Diffuse expression of cytokeratin (CK) (**A**) and chromogranin A (**B**) in a GB-NET of the gallbladder fundus; (**C**) Moderate-to-strong membranous expression of somatostatin receptor type 2A (SSTR2) in most neoplastic cells (Magnification, ×40 for all panels)

Immunohistochemical findings are summarized in Table 2. All tumors (26/26) showed diffuse expression of keratins (Fig. 4A) and synaptophysin, with diffuse chromogranin A positivity in all (Fig. 4B) but two cases. SSTR2A demonstrated strong, diffuse membranous staining (score 3+) in 3 of 6 tumors (50%) (Fig. 4C), weak focal staining (score 2+) in 2 (33%), and was negative in 1 (17%).

**Table 2:** Immunohistochemical Profiles of Gallbladder Neuroendocrine Tumors (GB-NETs) from the Multicenter Series

Immunohistochemical marker	Number of positive cases/number of tested cases (%)
Synaptophysin	26/26 (100%, diffuse)
Chromogranin A	26/26 (100%, diffuse in 24, focal in 2)
Pan-keratin (AE1/AE3)	26/26 (100%, diffuse)
Somatostatin receptor type 2 A	5/6 (83%, diffuse in 3, focal in 2)
ISLET1	5/5 (100%, diffuse)
ARX	5/5 (100%, diffuse)
CDX2	1/7 (14%, focal)
SATB2	2/6 (33%, focal)
TTF1	0/4
GATA3	0/6
Pancreatic polypeptide	5/7 (71%, diffuse in 1, focal in 4)
Somatostatin	5/8 (62.5%, diffuse in 3, focal in 2)
Gastrin	4/7 (57%, diffuse in 2, focal in 2)
Glucagon	1/5 (20%, focal)
Serotonin	0/4
Insulin	0/6
S100 protein	6/6 (100%, diffuse in 3, focal in 3)

**Fig. 5:**
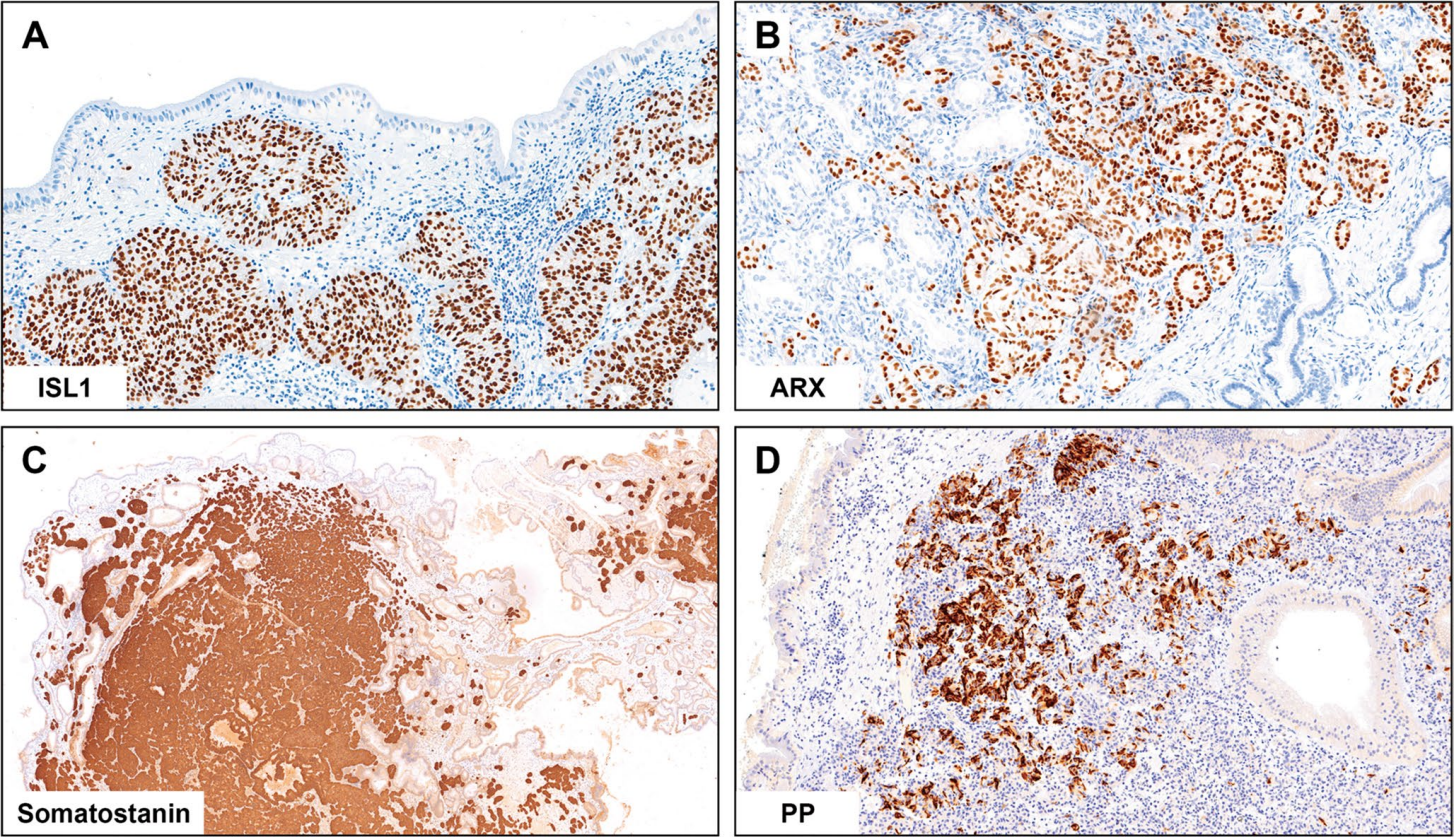
Transcription factor and hormone expression in GB-NETs. (**A**) ISLET1 (ISL1) and (**B**) ARX show diffuse expression; non-neoplastic gallbladder epithelium is negative for both transcription factors. (**C**) Somatostatin shows diffuse expression in a GB-NET of the gallbladder fundus (**D**) Pancreatic polypeptide (PP) shows focal expression. (Magnification, ×40 for all panels)

All tested tumors (*n* = 5) showed diffuse nuclear expression of ISLET1 and ARX (Fig. 5A, B), whereas GATA3 and TTF1 were uniformly negative. CDX2 and SATB2 were only rarely expressed, showing weak focal staining in 1 of 7 and 2 of 6 tumors, respectively.

Among hormonal markers, PP and somatostatin were most frequently expressed (PP: 5/7, 71%; somatostatin: 5/8, 62.5%) (Fig. 5C, D), followed by gastrin (4/7, 57%), which was confined to tumors of the neck/cystic duct, and glucagon (1/5, 20%). Serotonin and insulin were negative in all tested cases. Five tumors which were assessed for multiple hormones were predominantly positive for somatostatin and focally positive for PP, gastrin, and/or glucagon.

Diffuse somatostatin expression correlated with paraganglioma-like histology.

S100 (typically weak and focal) labeled tumor cells in all six tested cases, including tumors lacking a paraganglioma-like pattern. In tumors with a paraganglioma-like architecture, S100-positive sustentacular-like cells were occasionally accentuated at the periphery of tumor nests; however, the neoplastic cells themselves were also positive (Supplementary Fig. 2B); ganglioneuroma components were not seen.

### Comparison with GB-NECs

Compared to GB-NECs (Table 3), GB-NET patients had a lower age at diagnosis (50 vs. 58 years) and lower female-to-male ratio (1.9 vs. 5.2). Histologically, GB-NECs had higher rates of tumor necrosis and pT3/4 stage and lymph node metastasis. Importantly, 65% of GB-NECs patients died of disease, whereas no death occurred among GB-NET patients.

**Table 3:** Comparison of Clinicopathologic Features of GB-NETs and GB-NECs

	GB-NETs (*n* = 26)	GB-NECs (*n* = 31)*
Female/male ratio	17/9 (1.9:1)	26/5 (5.2:1)
Median patient age (range)	50 years (23–88)	58 years (26–93)
Ki67 proliferative index, median (range)	1% (0.5-8)	70% (40–95)
Tumor necrosis, n (%)	0	24 (89)
pT stage°		
pT1, n (%)	11 (42)	0
pT2, n (%)	15 (58)	8 (27)
pT3/T4, n (%)	0	22 (73)
pN stage°		
pN0, n (%)	8 (100)	5 (50)
pN1, n (%)	0	5 (50)
Tumor-related death, n (%)^	0	15 (65)

*data from Ref. 5, including 22 mixed neuroendocrine-non-neuroendocrine neoplasms (MiNENs) with a neuroendocrine carcinoma (NEC) component

*GB-NET gallbladder neuroendocrine tumor, GB-NEC gallbladder neuroendocrine carcinoma, WHO* World Health Organization, *ICPN* intracholecystic papillary-tubular neoplasm

°according the UICC staging system (9th ed.) of gallbladder carcinoma

^information available for only 13 patients

### Systematic Literature Review (Case-Report) Cohort of GB-NETs

The PRISMA flow diagram is shown in Supplementary Fig. 3 (Supplementary Material 3). Seventy publications were included (Supplementary Material 4), describing 79 GB-NETs in 78 patients. Forty-two cases originally classified as “carcinoids” and one case classified as “somatostatinoma” were reclassified as NETs according to the current WHO criteria.

Clinicopathologic data are summarized in Table 4. The female-to-male ratio was 1.05:1, and the median age was 52 years (range, 25–81). Three patients (4%) had hereditary tumor syndromes (2 MEN1; 1 von Hippel-Lindau). Functional tumors were rare (2 cases, 3%, both gastrin-positive and associated with Zollinger–Ellison syndrome, arising in the cystic duct/neck; one occurred in a patient with MEN1). Cholelithiasis occurred in 60% of patients.

**Table 4:** Clinicopathologic Features of Gallbladder Neuroendocrine Tumors (GB-NETs) from the Systematic Literature Review

Variable			*N*. cases with available data
Patient sex	Female: Male	40:38 (1.05:1)	78
Patient age at NET diagnosis	Median (range)	52 years (25–81)	78
NET size (cm)	Median (range)	1.3 cm (0.1–14)	66
Hormonal syndrome	Yes, n (%)	2 (3)	75
Hereditary tumor syndrome	Yes, n (%)	3 (4)	78
Cholelithiasis	Yes, n (%)	34 (60)	57
NET site	Gallbladder neck, n (%)	25 (39)	64
	Cystic duct, n (%)	17 (27)	
	Gallbladder fundus, n (%)	16 (25)	
	Gallbladder body, n (%)	3 (5)	
	Gallbladder body and fundus, n (%)	2 (3)	
	Gallbladder neck and body, n (%)	1 (2)	
WHO grade	G1, n (%)	25 (71.5)	35
	G2, n (%)	4 (11.5)	
	G3, n (%)	6 (17)	
Lymphovascular invasion	Yes, n (%)	8 (29)	28
Perineural invasion	Yes, n (%)	4 (31)	13
Hormonal expression by tumor cells	Somatostatin, n (%)	8 (61.5)	13
	Pancreatic polypeptide, n (%)	7 (70)	10
	Gastrin, n (%)	5 (38)	13
	Serotonin, n (%)	2 (20)	10
	Glucagon, n (%)	0	8
	Insulin, n (%)	0	7
Metastasis (lymph nodes or distant) at diagnosis	Yes, n (%)	19 (29)	66
Tumor-related death	Yes, n (%)	7 (11)	63

*NET* neuroendocrine tumor, *WHO *World Health Organization

The most common site was the gallbladder neck (*n* = 25, 39%). Median tumor size was 1.3 cm (range, 0.1–6.5). Among tumors with reported/assignable WHO grade, 71.5% (*n* = 25) were G1, 11.5% (*n* = 4) G2, and 17% (*n* = 6) G3. Lymphovascular invasion and perineural invasion were identified in 29% (*n* = 8) and 31% (*n* = 4) of cases, respectively.

Metastases at diagnosis were identified in 19 of 66 patients (29%) with available data: 7 had isolated cystic duct/loco-regional lymph node involvement and 12 had distant metastases, mainly to the liver. Among those with metastases and follow-up (*n* = 16), 44% (7 of 16) died of disease. Five of the seven had distant metastases and two had regional metastasis. Tumor size was available in six of these cases, five of which were larger than 2 cm; tumor grade was reported for one case (G3). None of the nonmetastatic patients (0 of 42) died of GB-NET.

In a small subset with immunohistochemical data available, somatostatin and PP were the most frequently expressed hormones, detected in 8/13 (61.5%) and 7/10 (70%) of examined cases, respectively.

### Clinicopathologic Features Stratified by Tumor Location

To assess site-related differences, GB-NETs were analyzed by tumor location in both cohorts (Table 5 and Supplementary Table 1/Material 5). In the literature cohort, body/fundus tumors (*n* = 21) were larger than neck/cystic duct tumors (*n* = 25; median, 2.5 vs. 1.2 cm; *p* = 0.014). All six G3 tumors occurred in the body/fundus, which also had higher rates of metastasis (40% vs. 12% for cystic duct/neck tumors; *p* = 0.039) and tumor-related mortality (*p* = 0.035).

**Table 5:** Clinicopathologic Features of Gallbladder Neuroendocrine Tumors (GB-NETs) from the Systematic Case Report Review, Stratified by Tumor Site

		Cystic duct/gallbladder neck GB-NETs (*n* = 42)	Body/Fundus GB-NETs (*n* = 21)	*p*-value
Male sex	*n* (%)	16/42 (38)	13/21 (62)	0.108
Patient age at NET diagnosis	Median (range)	51.5 (28–81)	51 (27–81)	0.795
NET size (cm)	Median (range)	1 (0.2-5)	2.5 (0.5–14)	**0.014**
Hereditary tumor syndrome	Yes, n (%)	2/42 (5)	0/21 (0)	0.548
Hormonal syndrome	Yes, n (%)	2/42 (5)	0/21 (0)	0.548
WHO grade	G1-G2, n (%)	18/18 (100)	7/13 (54)	**0.002**
	G3, n (%)	0/18 (0)	6/13 (46)	
Lymphovascular invasion	Yes, n (%)	3/14 (21)	3/11 (27)	1.000
Perineural invasion	Yes, n (%)	4/8 (50)	0/5 (0)	0.105
Cholelithiasis	Yes, n (%)	23/36 (64)	5/10 (50)	0.480
Hormonal expression	Somatostatin, n (%)	6/10 (60)	2/2 (100)	0.515
	Pancreatic polypeptide, n (%)	6/7 (86)	1/2 (50)	0.417
	Gastrin, n (%)	4/11 (36)	0/1 (0)	1.000
Metastasis	Yes, n (%)	4/34 (12)	8/20 (40)	**0.039**
Tumor-related death	Yes, n (%)	0/33 (0)	3/17 (18)	**0.035**

*GB-NET *gallabladder neuroendocrine tumor, *NET* neuroendocrine tumor, *WHO* World Health Organization

In our cohort, body/fundus tumors showed a male predominance (male-to-female ratio, 2:1) compared with neck/cystic duct tumors (1/13; *p* = 0.004). Tumor site also correlated with gross morphology: 10 of 14 polypoid tumors arose in the body/fundus, whereas 10 of 12 flat or nodular tumors arose in the neck/cystic duct (*p* = 0.005). No significant differences were observed for age, hereditary syndromes, tumor size, WHO grade, pT/pN stage, or other associated gallbladder pathology. Notably, cholesterol-related lesions appeared to show a trend: cholesterol polyps were more frequently found in the body/fundus (6 cases) than in the neck (1 case), and cholesterolosis was observed in 50% of body/fundus tumors vs. 23% of neck/cystic duct tumors. Although these differences did not reach statistical significance (*p* = 0.226), this is likely due to the limited number of cases. In the literature, only one case of GB-NET associated with cholesterolosis [20] and another associated with a cholesterol polyp [21] have been reported.

### Comparison of Clinicopathologic Features Between Nonmetastatic and Metastatic GB-NETs

As shown in Table 6, metastatic tumors at diagnosis identified in literature (*n* = 19) were significantly larger (median tumor size: 3.0 cm) compared with non-metastatic tumors (median tumor size: 1.0 cm in the non-metastatic literature cohort (*n* = 47; *p* = 0.002) and 0.9 cm in the pooled non-metastatic group including our series (*n* = 72; *p* < 0.001). Tumors > 2 cm were more common in metastatic cases (69%) than in the literature non-metastatic cohort (19%; *p* = 0.001) or the pooled non-metastatic group (11%; *p* < 0.001). High-grade (WHO G3) NETs were enriched among metastatic tumors (67%) but were rare in non-metastatic cases (8% in the literature non-metastatic cohort and 4% in the pooled non-metastatic group; *p* = 0.006 and *p* < 0.001, respectively). Metastatic tumors also more frequently arose in the body/fundus (67% vs. 29% in the literature non-metastatic cohort and 35% in the pooled non-metastatic group; *p* = 0.039 and *p* = 0.055).

**Table 6:** Clinicopathologic Factors Associated with Metastatic Gallbladder Neuroendocrine Tumors (GB-NETs)

		Metastatic GB-NETs at diagnosis (*n* = 19) Group A	Non-metastatic GB-NETs from literature review (*n* = 47) Group B	*p*-value (A vs. B)	Combined non-metastatic GB-NETs from literature review and case series (*n* = 72) Group C	*p*-value (A vs. C)
NET size, cm	Median (range)	3 (0.2–14)	1 (0.2–6.5)	0.002	0.9 (0.2–6.5)	< 0.001
NET size > 2 cm	n (%)	9/13 (69)	8/43 (19)	**0.001**	8/72 (11)	**< 0.001**
WHO grade G3	n (%)	4/6 (67)	2/26 (8)	**0.006**	2/51 (4)	**< 0.001**
Body/fundus tumor site	n (%)	8/12 (67)	12/42 (29)	**0.039**	23/66 (35)	0.055

*GB-NET* gallbladder neuroendocrine tumor, *NET* neuroendocrine tumor, *NS* not significant, *WHO W*orld Health Organization

## Discussion

This study, which is the largest and most comprehensive clinicopathologic analysis of GB-NETs to date, brings numerous new perspectives and clarifications to the clinical, pathologic, immunophenotypic and behavioral characteristics of GB-NETs, including clinical, histology, immunohistochemical characteristics, outcome of the patients and possible cell origin.

### Clinical Characteristics

Foremost, our findings highlight that non-metastatic GB-NETs are a more indolent tumor group than has been thought before. Earlier literature describing GB-NETs as uniformly aggressive often failed to distinguish NETs from NECs; for example, a SEER 1973–2005 study reported a median survival of 10 months for “gallbladder NETs” [3], probably reflecting the inclusion of NECs. However, studies applying more rigorous analyses using the updated diagnostic criteria have demonstrated markedly different outcomes. Zhou et al., using the SEER 2000–2017 database, reported median survivals of 79 months for GB-NETs and 11 months for GB-NECs [22]. Notably, the survival of patients with extrahepatic bile duct NET (78 months) was similar to that of GB-NET patients.

Consistent with their indolent nature, tumors in our cohort exhibited non-aggressive morphologic features. They were typically small at the time of diagnosis (median size, 0.6 cm), with minimal infiltration and very low proliferative activity; approximately 80% were classified as G1. Most tumors were superficial (pT1 or very limited pT2). Considering they are incidental tumors, it is reasonable to assume that their growth prevents them from reaching a substantial size, which is consistent with their low Ki-67 index. Lymphatic and vascular invasions were rare, and perineural invasion, when present, was limited to intratumoral entrapped nerves. None was grade G2b [11] or G3, all were non/minimally infiltrative per the recent grading system proposed for Pan-NETs [15], and some showed the morphologic patterns (paraganglioma-like and symplastic/pleomorphic) associated with more benevolent behavior and none had patterns linked to aggressive behavior [13]. Importantly, no patients presented with metastatic disease and, during a median follow-up of 30 months, no recurrences or tumor-related deaths occurred. These features are in stark contrast with GB-NECs which are very aggressive malignancies. In a separate analysis by some of the authors of this study, NECs were found to have very aggressive behavior with a median survival of 6 months, very high Ki-67 index (median, 70%), advanced tumors at the time of diagnosis, and high rates of perineural, lymphatic and/or vascular invasion [5].

We also conducted a systematic review of the literature. It was clear that in many studies NECs and NETs were reported together. We tried our best to clean out a cohort of publications that appeared to represent true NETs (not NECs and other tumors) and this group highlighted the following findings: “GB-NETs” behaved more aggressively if they had metastatic disease at the time of diagnosis. Of note, none of our cases had metastatic disease. Additionally, all GB-NET patients who died of disease had metastatic NETs already at diagnosis, and 44% of patients with metastatic GB-NET died of disease. The relatively larger tumor size (median: 1.3 cm), as well as the higher frequency of G3 cases (17%) and of locoregional and/or distant metastatic disease (29%) extracted from published case reports should be interpreted with caution due to potential publication bias favoring the reporting of rarer and more aggressive cases. Furthermore, in the G3 NETs reported in the literature, it is not always possible to definitively exclude the possibility that these cases may actually represent NECs, as the available images are often limited and immunohistochemical work-up is not consistently provided (e.g., p53 and Rb immunohistochemistry). Literature indicates that tumor grade G3 (which, before 2017 also officially included NECs per WHO classification) and tumor size > 2 cm were predictors of metastatic potential in GB-NETs. Although our multinational series did not show any metastatic case, considering the fact that all NETs are considered malignant, patients with GB-NETs should undergo comprehensive staging using multiple imaging modalities and be closely monitored. Moreover, although clear evidence is lacking, extrapolation from the treatment of NETs at other sites suggests that radical cholecystectomy with lymphadenectomy could be considered in cases with more advanced tumors (≥ T2), those larger than 2 cm, or with adverse pathologic findings like perineural and lymphatic or vascular invasion. In terms of size, 2 cm cut-off appears to be a reasonable threshold, based not only on our data and the available literature on GB-NETs (albeit, the latter not very reliable), but also on evidence from NETs of pancreas and appendix where a < 2 cm cut-off is now widely used to guide the decision on whether radical operation is warranted [23].

### Morphologic Variants and Immunohistochemical Features

The morphologic spectrum of GB-NETs in this study is broader than so far reported. Most tumors exhibited classic nested or trabecular architecture; however, additional patterns included paraganglioma-like areas, symplastic-type degenerative changes, and pseudoglandular patterns, with or without psammoma bodies. Paraganglioma-like and degenerative features, associated with indolent behavior in Pan-NETs [13], were observed in 38% of cases, usually focally. Although these features can mimic true paragangliomas, GB-NETs consistently express keratins and lack GATA3, distinguishing them from the exceedingly rare gallbladder paragangliomas. Also, not surprisingly, as is the case in the pancreas, some of these paraganglioma-like GB-NETs also had substantial symplastic pleomorphic cells. Less common histologic variants included spindle cell tumors, sclerohyaline stromal changes, and clear cell alterations. None had oncocytic, hepatoid or diffuse growth patterns. Clear cell changes have been reported in biliary, pancreatic and ampullary NETs associated with von Hippel-Lindau syndrome [24, 25], though the three cases in our series were likely sporadic. Overall, genetic syndromes appear uncommon, reported in approximately 4% of cases and represented in our cohort by a single MEN1-associated tumor (without Zollinger-Ellison syndrome), noting that germline testing was not performed uniformly.

Immunohistochemical analysis further clarifies GB-NET biology and potential lineage relationships. SSTR2A expression was common (> 70%), offering both diagnostic and therapeutic utility through somatostatin-receptor–based imaging and identifying candidates for somatostatin analog therapy or peptide receptor radionuclide therapy. Among hormonal markers, PP and somatostatin were most frequently expressed, with somatostatin correlating with paraganglioma-like morphology, as reported in Pan-NETs [13]. Gastrin expression was restricted to tumors of the gallbladder neck and cystic duct and was associated with Zollinger-Ellison syndrome in two cases (from the literature).

Transcription factor analysis revealed diffuse ISLET1 and ARX expression, supporting a foregut-type neuroendocrine differentiation profile and suggesting affinity with alpha-cell or PP-cell lineage programs described in pancreatic NETs [26, 27]. The combination of strong ARX expression and PP positivity in 71% of tumors further points to a potential lineage relationship with Pan-NETs, although additional molecular studies are needed to confirm this assumption. S100 expression was also common, which could potentially lead to diagnostic confusion with paragangliomas or composite gangliocytoma/neuroma and neuroendocrine tumour (CoGNET), particularly in tumors with paraganglioma-like morphology, emphasizing the importance of combining cytokeratin and neuroendocrine markers for accurate diagnosis. Moreover, it should be noted that S100-positive sustentacular and/or neuroendocrine tumor cells may also be encountered in other NET subsets, such as pancreatic, ampullary, appendiceal, and lung NETs [28–30].

In our study we were only able to conduct such examinations in a limited number of cases due to either the lack of available material (most cases were second opinion consults) or small size of tumor disallowing comprehensive immunohistochemical analysis.

### Regional Associations and Possible Cell of Origin

In this study we found that GB-NETs have some different presentations and characteristics based on the location in the body/fundus versus neck/cystic-duct region, and these may potentially have some biologic significance. In our cohort, body/fundus tumors showed a male predominance and were more frequently polypoid, while published cases additionally demonstrated larger size, higher rates of G3 grade, and more frequent metastasis. By contrast, neck/cystic duct tumors were typically smaller mural nodules with a female predominance.

The histogenesis of GB-NETs remains debated. Neck/cystic duct NETs may arise from dispersed neuroendocrine cells within mucous gland epithelium [3, 17]. However, as the normal epithelium of the gallbladder body and fundus appears to lack neuroendocrine cells [31], the development of GB-NETs in these regions is difficult to explain; consistent with this, we also did not observe chromogranin A-positive epithelial neuroendocrine cells in the non-metaplastic, non-dysplastic mucosa of the gallbladder body-fundus distant from the neck/cystic duct NET in some cases in which such mucosa was present in the same histologic block as the NET. One proposed mechanism is inflammation-induced epithelial metaplasia, capable of producing somatostatin-, gastrin-, and PP-producing cells [31, 32]. However, metaplasia was present in only one-third of our cases, and cholelithiasis in less than half, also seems to fail in explaining the origin of all NETs arising in the body and fundus of the gallbladder. Heterotopic pancreatic tissue has also been proposed as a source [33], though no heterotopic pancreatic tissue was identified in any of our cases, and therefore, other mechanisms may be involved, including an origin from multipotent stem cells within the gallbladder mucosa.

### Role of Cholesterolosis and Cholesterol Polyps

An interesting speculative observation in our case series, potentially relevant to etiopathogenesis, is the association of GB-NETs with cholesterolosis, as 50% of body/fundus tumors were associated with cholesterolosis, exceeding the 6.5–24% prevalence reported in unselected cholecystectomy specimens [34, 35]. In many case reports of GB-NETs, the description of the background gallbladder mucosa in which the tumor arises is limited, which may account for the discrepancy between our findings and those reported in the literature regarding a potential association between neuroendocrine tumors and cholesterolosis/cholesterol polyps in the literature. Even more intriguingly, several cases were arising in cholesterol polyps, which showed both the pathognomonic cauliflower-like architecture of cholesterol polyps, as well as the presence of cholesterolosis within the polyps. These polyps typically arise in relatively young individuals (mean age: 46 years), with a female-to-male ratio of 2.2. They are one of the few pathologic conditions in the gallbladder that appear to be almost as common in males as in females, particularly in Western countries [19]. The proposed pathogenic association between a subset of GB-NETs and cholesterol polyps could help explain the relatively high frequency of body/fundus GB-NETs in male patients observed in our study, as well as their relatively young age at onset. Intracholecystic tubular non-mucinous neoplasm (ICTN), a peculiar invasion-resistant type of mass-forming intracholecystic neoplasm composed of small tubular units with minimal/no cytoplasm and complex architecture, has been reported to arise from cholesterol polyps [36]. Interestingly, ICTNs may also frequently (39%) exhibit subtle neuroendocrine cell clusters intermingled with dysplastic glandular cells [36]. Therefore, although cholesterol polyps likely arise in a microenvironment that seems to be protective against development of ordinary gallbladder adenocarcinomas, the chemical milieu of cholesterolosis may promote the induction of neuroendocrine proliferations. Several speculative mechanisms may explain this association. Cholesterol is required for activation of the Hedgehog (Hh) pathway, and aberrant Hh signaling has been proposed as a driver of NET development by promoting progenitor cell self-renewal and inhibiting apoptosis [37, 38]. Excess cellular cholesterol may also increase membrane lipid rafts, amplifying signaling through G-protein coupled receptors and growth factor receptors, while lipid-derived molecules (e.g., oxysterols) may stimulate neuroendocrine precursor cells via paracrine loops [39, 40]. These mechanisms remain entirely speculative at this time, but may provide a basis for future investigation. Specifically, future studies evaluating cholesterol polyps with neuroendocrine immunohistochemical markers may help further elucidate their potential biological relationship with GB-NETs.

### Limitations

The retrospective, multinational design of this study introduces inherent case selection variability and incomplete clinical data. In addition, many cases were referred as second-opinion consultations to expert centers, which may further contribute to selection bias. Follow-up data were available only for a subset of patients, limiting the assessment of long-term behavior. These limitations underscore the need for future studies with more comprehensive and standardized data collection. In particular, incorporating detailed analyses of transcription factors, hormone expression profiles, and molecular features may provide further insights into the biology of these tumors and help refine their diagnostic classification. Such studies will also be essential to clarify the relationship between GB-NETs and NETs arising at other anatomical sites, as well as to further delineate potential tumorigenic pathways suggested by our findings. Finally, the limitations of the systematic review should also be also acknowledged, including the absence of the use of Scopus in the literature search strategy.

## Conclusions

This study, which is the most extensive analysis of GB-NETs to date, demonstrates that GB-NETs represent a distinct group of digestive NETs characterized by morphological features such as paraganglioma-like pattern (associated with somatostatin hormone positivity immunohistochemically) and symplastic degenerative cellular features as well as a predominant expression of PP and somatostatin combined with diffuse ISLET1 and ARX expression. Gastrin positive tumors are third in frequency and appear to be mainly localized in neck and cystic duct regions. NETs arising in the gallbladder body and fundus occur more frequently in males (in contrast to most gallbladder pathologies, which typically show a female-to-male ratio of ≥ 3:1), are more often polypoid and more commonly exhibit features associated with aggressive behavior compared to those in the cystic duct or neck, thus warranting greater clinical attention. The notable association of GB- NETs arising in the gallbladder proper with cholesterolosis and cholesterol polyps is interesting in view of a link to a pathogenetic mechanism underlying this observation. However, this has to be studied in a higher number of cases. Importantly, our findings may help inform the clinical management of these exceptionally rare tumors.

## Electronic Supplementary Material

Below is the link to the electronic supplementary material.

Supplementary Material 1 (PDF 379 KB

Supplementary figure 2 Entrapped nerve in a gallbladder neuroendocrine tumor (GB-NET): (A) H&E, magnification 100x; (B) S100 immunohistochemical stain highlighting the nerve, magnification 100x. (PNG 6.77 MB)

High Resolution Image (TIFF 15.4 MB)

Supplementary Material 3 (PDF 79.1 KB)

Supplementary Material 4 (PDF 248 KB)

Supplementary Material 5 (PDF 512 KB)

## Data Availability

The data that support the findings of this study are not openly available due to reasons of sensitivity and are available from the corresponding author upon reasonable request.
