# Supplementary material for: Neuroendocrine Tumors of the Gallbladder: A Multicenter Case Series and Systematic Literature Review Indicating Predominantly Non-Aggressive Tumor Behavior and a Common Association with Cholesterol Polyps and Cholesterolosis
Source: Endocr Pathol. 2026 Jun 11;37(1):26. doi: 10.1007/s12022-026-09921-3 (PMC13260158; doi:10.1007/s12022-026-09921-3)
Supplement: Supplementary file 1 — Supplementary Material 1 (PDF 379 KB [file 12022_2026_9921_MOESM1_ESM.pdf]

Date	Database	Search query	Search database
18th April 2025	**Pubmed**	("Gallbladder"[Mesh] OR gallbladder[Title/Abstract] OR "gall bladder"[Title/Abstract] OR "Gallbladder Neoplasms"[Mesh] OR "Gallbladder Neoplasms"[Title/Abstract] OR "Cystic Duct"[Mesh] OR "cystic duct"[Title/Abstract]) AND ("Neuroendocrine Tumors"[Mesh] OR "neuroendocrine tumor*"[Title/Abstract] OR "neuroendocrine neoplasm*"[Title/Abstract] OR "Carcinoid Tumor"[Mesh] OR carcinoid*[Title/Abstract])	English, Humans, Sort by: Most Recent
18th April 2025	**Embase**	('gallbladder'/exp OR 'gallbladder':ti,ab,kw OR 'gall bladder':ti,ab,kw OR 'gallbladder tumor'/exp OR 'gallbladder neoplasms':ti,ab,kw OR 'cystic duct'/exp OR 'cystic duct':ti,ab,kw) AND ('neuroendocrine tumor'/exp OR 'neuroendocrine tumor*':ti,ab,kw OR 'neuroendocrine neoplasm*':ti,ab,kw OR 'carcinoid'/exp OR 'carcinoid*':ti,ab,kw)	Limits: ADN [english]/lim AND ('article'/it OR 'article in press'/it OR 'review'/it)

Supplementary Figure 1. Search databases and queries for systematic literature review on gallbladder/cystic duct neuroendocrine tumors.
