# Supplementary figures and images for: Neuroendocrine Tumors of the Gallbladder: A Multicenter Case Series and Systematic Literature Review Indicating Predominantly Non-Aggressive Tumor Behavior and a Common Association with Cholesterol Polyps and Cholesterolosis
Source: Endocr Pathol. 2026 Jun 11;37(1):26. doi: 10.1007/s12022-026-09921-3 (PMC13260158; doi:10.1007/s12022-026-09921-3)

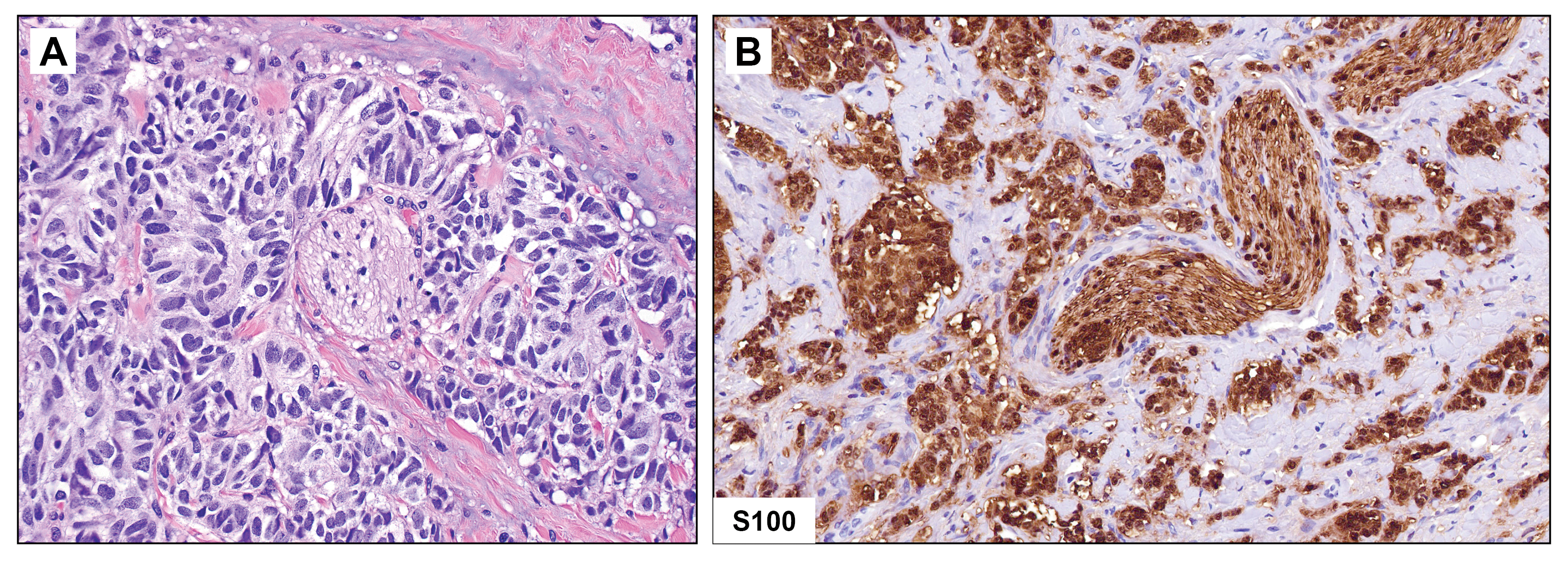

Supplement: Supplementary file 2 — Entrapped nerve in a gallbladder neuroendocrine tumor (GB-NET): (A) H&E, magnification 100x; (B) S100 immunohistochemical stain highlighting the nerve, magnification 100x. (PNG 6.77 MB) [file 12022_2026_9921_Fig6_ESM.png]

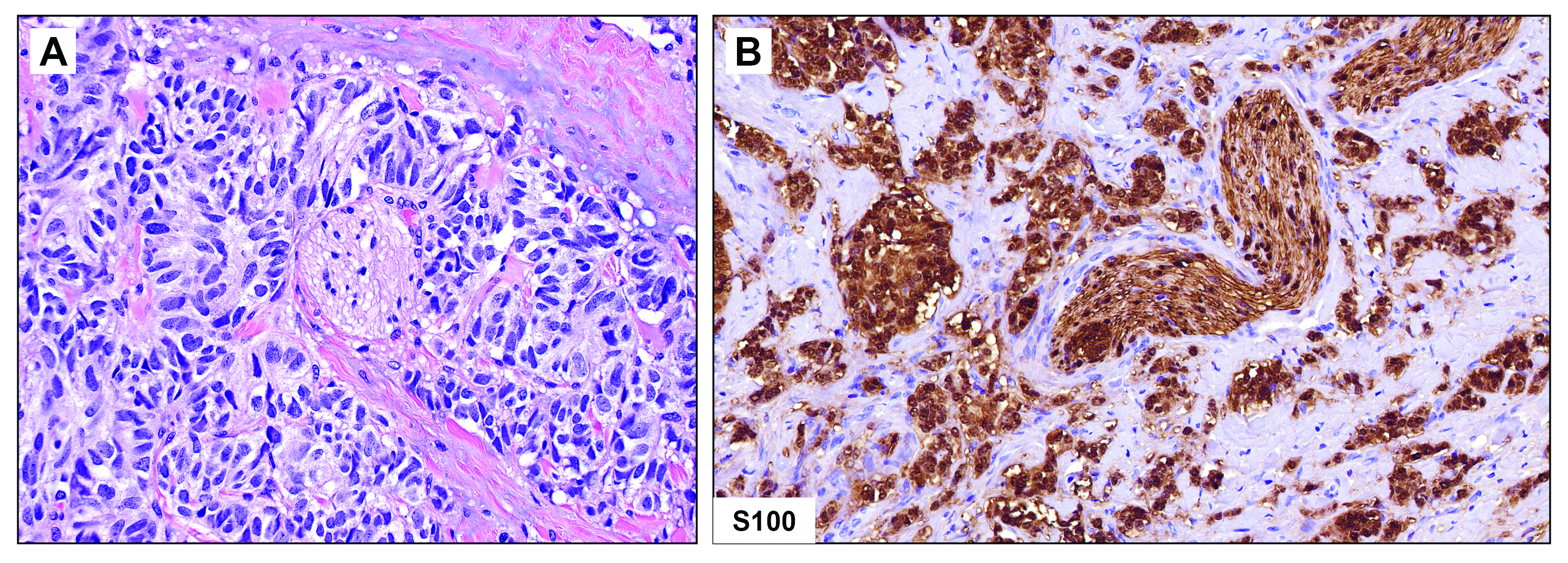

Supplement: Supplementary file 3 — High Resolution Image (TIFF 15.4 MB) [file 12022_2026_9921_MOESM2_ESM.tif]
