# Supplementary material for: Neuroendocrine Tumors of the Gallbladder: A Multicenter Case Series and Systematic Literature Review Indicating Predominantly Non-Aggressive Tumor Behavior and a Common Association with Cholesterol Polyps and Cholesterolosis
Source: Endocr Pathol. 2026 Jun 11;37(1):26. doi: 10.1007/s12022-026-09921-3 (PMC13260158; doi:10.1007/s12022-026-09921-3)

Supplementary Figure 3. PRISMA 2020 flow diagram for the systematic literature review on gallbladder/cystic duct well-differentiated neuroendocrine tumors

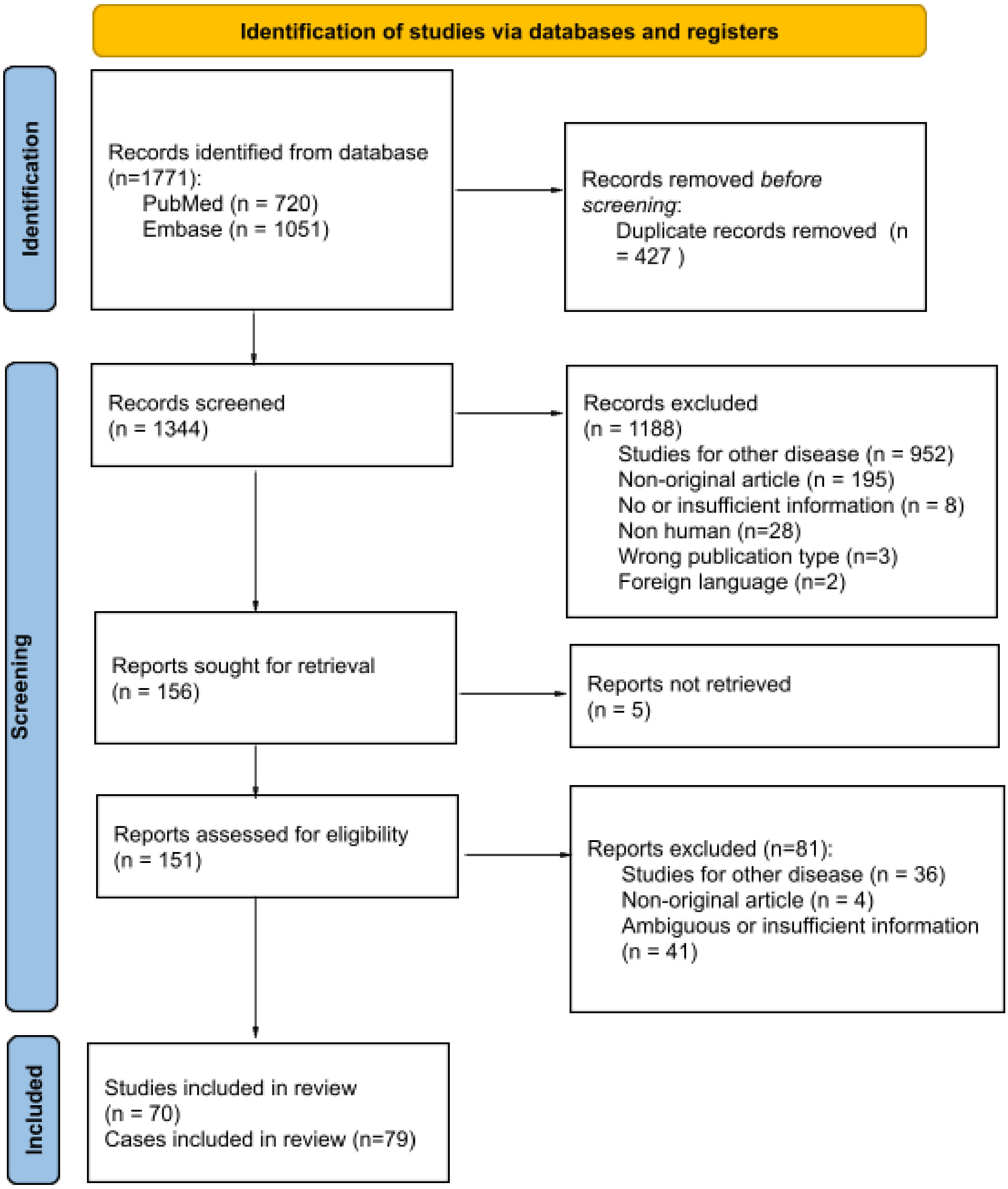

Supplement: Supplementary file 4 — Supplementary Material 3 (PDF 79.1 KB) [file 12022_2026_9921_MOESM3_ESM.pdf]
