# Supplementary material for: Neuroendocrine Tumors of the Gallbladder: A Multicenter Case Series and Systematic Literature Review Indicating Predominantly Non-Aggressive Tumor Behavior and a Common Association with Cholesterol Polyps and Cholesterolosis
Source: Endocr Pathol. 2026 Jun 11;37(1):26. doi: 10.1007/s12022-026-09921-3 (PMC13260158; doi:10.1007/s12022-026-09921-3)
Supplement: Supplementary file 5 — Supplementary Material 4 (PDF 248 KB) [file 12022_2026_9921_MOESM4_ESM.pdf]

Studies included in the systematic review on gallbladder neuroendocrine tumors

1. Angelini C, Mussi C, Crippa S, Sartori P, Isimbaldi G, Bonardi C, Uggeri F. Gallbladder carcinoid: a case report. Chir Ital. 2003 Jul-Aug;55(4):571-3. PMID: 12938605.
2. Aronsky D, Z'graggen K, Stauffer E, Lange J, Klaiber C. Primary neuroendocrine tumors of the cystic duct. Digestion. 1999 Sep-Oct;60(5):493-6. doi: 10.1159/000007696. PMID: 10473975.
3. Ayub F, Saif MW. Neuroendocrine Tumor of the Cystic Duct: A Rare and Incidental Diagnosis. Cureus. 2017 Oct 6;9(10):e1755. doi: 10.7759/cureus.1755. PMID: 29226045; PMCID: PMC5718876.
4. BARNES TG. Argentaffinoma (carcinoid) of the gall bladder; a case report. Surgery. 1952 Oct;32(4):723-7. PMID: 12984292.
5. Barone GW, Schaefer RF, Counce JS, Eidt JF. Gallbladder and gastric argyrophil carcinoid associated with a case of Zollinger-Ellison syndrome. Am J Gastroenterol. 1992 Mar;87(3):392-4. PMID: 1539581.
6. Berman AJ, Schandler GT, Walton DB. Examination of Gallbladders at Military Treatment Facilities: Is Histologic Analysis Necessary? Mil Med. 2025 Jun 30;190(7-8):e1524-e1528. doi: 10.1093/milmed/usaf024. PMID: 39847129.
7. Chittal SM, Ra PM. Carcinoid of the cystic duct. Histopathology. 1989 Dec;15(6):643-6. doi: 10.1111/j.1365-2559.1989.tb01632.x. PMID: 2606458.
8. Dammak N, Ammar H, Latifa MB, Belkacem O, Mabrouk MB, Ali AB. Gallbladder primary well-differentiated neuroendocrine tumor: A misdiagnosed case. Int J Surg Case Rep. 2023 Oct;111:108882. doi: 10.1016/j.ijscr.2023.108882. Epub 2023 Oct 1. PMID: 37793232; PMCID: PMC10551650.
9. Deehan DJ, Heys SD, Kernohan N, Eremin O. Carcinoid tumour of the gall bladder: two case reports and a review of published works. Gut. 1993 Sep;34(9):1274-6. doi: 10.1136/gut.34.9.1274. PMID: 8406168; PMCID: PMC1375470.
10. Dhakre VW, Purushothaman G, Doctor N. Gallbladder Neuroendocrine Tumors: Is There a Need for a Specific Approach? Gastrointest Tumors. 2021 Nov 18;9(1):5-11. doi: 10.1159/000520988. PMID: 35528747; PMCID: PMC9021648.
11. Felekouras E, Petrou A, Bramis K, Prassas E, Papaconstantinou I, Dimitriou N, Pazaiti A, Tsigris C, Giannopoulos A. Malignant carcinoid tumor of the cystic duct: a rare cause of bile duct obstruction. Hepatobiliary Pancreat Dis Int. 2009 Dec;8(6):640-6. PMID: 20007084.
12. Frometa A, Chaudhary Y, Ansari O, Jagoo N, Kapadia I. Neuroendocrine Tumor of the Gallbladder, a Rare Incidental Finding. HCA Healthc J Med. 2020 Jun 27;1(3):149-154. doi: 10.36518/2689-0216.1009. PMID: 37424717; PMCID: PMC10324707.
13. Gaffney PR, Coyle LJ. Carcinoid tumour of the gall bladder associated with a meningioma. Ir J Med Sci. 1978 Sep;147(9):318-21. doi: 10.1007/BF02939426. PMID: 700976.
14. Garland J, O'Leary E, Haggerty J, Zorc TG. Carcinoid tumor of the cystic duct: case report, literature review and comparison of surgical approaches. Case Rep Oncol. 2014 Sep 3;7(3):621-4. doi: 10.1159/000367648. PMID: 25408653; PMCID: PMC4209269.
15. Geo SK, Harikumar R, Kumar S, Kumar B, Gopinath A. Gall bladder carcinoid: a case report and review of literature. Trop Gastroenterol. 2007 Apr-Jun;28(2):72-3. PMID: 18050844.
16. Goodman ZD, Albores-Saavedra J, Lundblad DM. Somatostatinoma of the cystic duct. Cancer. 1984 Feb 1;53(3):498-502. doi: 10.1002/1097-0142(19840201)53:3<498::aid-cncr2820530321>3.0.co;2-4. PMID: 6318950.

17. Hermina M, Starling J, Warner TF. Carcinoid tumor of the cystic duct. Pathol Res Pract. 1999;195(10):707-9; discussion 710. doi: 10.1016/S0344-0338(99)80063-6. PMID: 10549035.
18. Heymann MF, Fiche M, Dubois-Gordeeff A, Chetritt J, Cloarec D, Guiberteau B, Le Bodic MF. Endocrine cell carcinoma (carcinoid tumour) of the gallbladder producing pancreatic polypeptide and somatostatin. Histopathology. 1997 Jun;30(6):606-7. PMID: 9205872.
19. Hirose Y, Sakata J, Endo K, Takahashi M, Saito R, Imano H, Kido T, Yoshino K, Sasaki T, Wakai T. A 0.8-cm clear cell neuroendocrine tumor G1 of the gallbladder with lymph node metastasis: a case report. World J Surg Oncol. 2018 Jul 23;16(1):150. doi: 10.1186/s12957-018-1454-y. PMID: 30037336; PMCID: PMC6057040.
20. Hong N, Kim HJ, Byun JH, Kim SY, Kim KW, Kim JH, Hong SM. Neuroendocrine neoplasms of the extrahepatic bile duct: radiologic and clinical characteristics. Abdom Imaging. 2015 Jan;40(1):181-91. doi: 10.1007/s00261-014-0191-0. PMID: 25008023.
21. Hsiao TH, Wu CC, Tseng HH, Chen JH. Synchronous but separate neuroendocrine tumor and high-grade dysplasia/adenoma of the gall bladder: A case report. World J Clin Cases. 2022 Mar 6;10(7):2322-2329. doi: 10.12998/wjcc.v10.i7.2322. PMID: 35321155; PMCID: PMC8895191.
22. Hubalewska-Dydejczyk A, Trofimiuk M, Sowa-Staszczak A, Gilis-Januszewska A, Baczyńska E, Szybiński P, Anielski R, Matłok M, Bonicki W, Kunikowska J. Neuroendocrine tumours of rare location. Endokrynol Pol. 2010 May-Jun;61(3):322-7. PMID: 20602309.
23. Ichimaru C, Ishii T, Katanuma A. G1-Neuroendocrine tumor of gallbladder with unique dendritic morphology. J Hepatobiliary Pancreat Sci. 2022 Oct;29(10):e100-e103. doi: 10.1002/jhbp.1153. Epub 2022 May 5. PMID: 35510353.
24. Ioannidis O, Cheva A, Paraskevas G, Chatzopoulos S, Kotronis A, Papadimitriou N, Konstantara A, Makrantonakis A, Kakoutis E. Neuroendocrine tumor of the cystic duct. Acta Gastroenterol Belg. 2012 Sep;75(3):357-60. PMID: 23082709.
25. Ishida M, Shiomi H, Naka S, Tani T, Okabe H. Clear cell neuroendocrine tumor G1 of the gallbladder without von Hippel-Lindau disease. Oncol Lett. 2012 Dec;4(6):1174-1176. doi: 10.3892/ol.2012.899. Epub 2012 Sep 6. PMID: 23205114; PMCID: PMC3506718.
26. Iwanaga N, Sugo H, Noro T, Watanobe I, Ogura K. A Case of a Grade 3 Gallbladder Neuroendocrine Tumor With Rapid Recurrence After Curative Resection. Cureus. 2023 Oct 17;15(10):e47193. doi: 10.7759/cureus.47193. PMID: 38021717; PMCID: PMC10652657.
27. Kaiho T, Tanaka T, Tsuchiya S, Miura M, Saigusa N, Yanagisawa S, Takeuchi O, Kitakata Y, Saito H, Shimizu A, Miyazaki M. A case of classical carcinoid tumor of the gallbladder: review of the Japanese published works. Hepatogastroenterology. 1999 Jul-Aug;46(28):2189-95. PMID: 10521965.
28. Kanakala V, Kasaraneni R, Smith DA, Goulbourne IA. Primary neuroendocrine neoplasm of the gallbladder. BMJ Case Rep. 2009;2009:bcr12.2008.1352. doi: 10.1136/bcr.12.2008.1352. Epub 2009 May 25. PMID: 21686357; PMCID: PMC3029781.
29. Khetan N, Bose NC, Arya SV, Gupta HO. Carcinoid tumor of the gallbladder: report of a case. Surg Today. 1995;25(12):1047-9. doi: 10.1007/BF00311691. PMID: 8645939.
30. Kitagawa K, Takashima T, Matsui O, Kadoya M, Haratake KJ, Tsuji M. Angiographic findings in two carcinoid tumors of the gallbladder. Gastrointest Radiol. 1986;11(1):51-5. doi: 10.1007/BF02035032. PMID: 2417907.
31. Ko A, MacKenzie M, Chiu K, Yap WW, Melich G, MacKenzie S. Primary gallbladder neuroendocrine neoplasm: A case report of grade 1 well-differentiated neuroendocrine tumor. Int J Surg Case Rep. 2024 Dec;125:110604. doi: 10.1016/j.ijscr.2024.110604. Epub 2024 Nov 15. PMID: 39561576; PMCID: PMC11615934.

32. Koizumi M, Sata N, Kasahara N, Morishima K, Kaneda Y, Fujiwara T, Ota M, Hyodo M, Yasuda Y. Carcinoid tumor of the gallbladder: report of two cases. Clin J Gastroenterol. 2011 Oct;4(5):323-330. doi: 10.1007/s12328-011-0242-9. Epub 2011 Jul 23. PMID: 26189633.
33. Konishi E, Nakashima Y, Smyrk TC, Masuda S. Clear cell carcinoid tumor of the gallbladder. A case without von Hippel-Lindau disease. Arch Pathol Lab Med. 2003 Jun;127(6):745-7. doi: 10.5858/2003-127-745-CCCTOT. PMID: 12741904.
34. Kumar A, Kumar B, Muthu GS, Mitra S. 68Ga-DOTANOC PET/CT Detects a Rare Case of Metastatic Neuroendocrine Neoplasm of the Gallbladder. Clin Nucl Med. 2022 Jun 1;47(6):539-540. doi: 10.1097/RLU.0000000000004142. Epub 2022 Apr 5. PMID: 35384899.
35. Kumar S, Agarwal S, Bhargava SK, Minocha VR. Malignant carcinoid tumor of the gallbladder: a case report and review of literature. Trop Gastroenterol. 1992 Apr-Jun;13(2):78-84. PMID: 1413104.
36. Lee JH, Lee KG, Oh YH, Paik SS, Park HK, Lee KS. Carcinoid tumors of the extrahepatic biliary tract: report of four cases. Surg Today. 2011 Mar;41(3):430-5. doi: 10.1007/s00595-010-4256-6. Epub 2011 Mar 2. PMID: 21365432.
37. Lim HU, Chan CC, Knotts FB. An incidental finding of carcinoid tumor of the cystic duct. J Surg Case Rep. 2013 Apr 4;2013(4):rjt021. doi: 10.1093/jscr/rjt021. PMID: 24964431; PMCID: PMC3635152.
38. Liu Y, Esnakula AK, Jain S, Lin J, Panarelli N, Pyatibrat S, Karamchandani DM. Spectra of well-differentiated neuroendocrine lesions in the extrahepatic biliary system: a case series. Histopathology. 2025 Jan;86(2):285-293. doi: 10.1111/his.15316. Epub 2024 Sep 12. PMID: 39267205.
39. Liu YG, Jiang ST, Zhou Y, Zhang JW, Sang XT, Zhang L, Lu X, Xu YY. Primary gastrinoma of the gallbladder: a case report and review of the literature. Front Oncol. 2024 Jan 31;13:1279766. doi: 10.3389/fonc.2023.1279766. PMID: 38357423; PMCID: PMC10864482.
40. Loharkar S, Basu S. Grade 3 metastatic neuroendocrine neoplasms of two unusual primary sites with contrasting differentiation characteristics: Dual tracer positron emission tomography and computed tomography imaging (^{18}F-fluorodeoxyglucose and ^{68}Ga-DOTATATE) correlates and their treatment implications. World J Nucl Med. 2020 Sep 9;20(1):125-128. doi: 10.4103/wjnm.WJNM_67_20. PMID: 33850504; PMCID: PMC8034780.
41. López Marcano JA, Ramia Ángel JM, de la Plaza Llamas R, Manuel Vázquez A, Latorre Fragua R. Neuroendocrine tumour of the gallbladder. Gastroenterol Hepatol. 2020 Feb;43(2):87-88. English, Spanish. doi: 10.1016/j.gastrohep.2019.09.003. Epub 2019 Nov 19. PMID: 31757444.
42. Machado MC, Penteado S, Montagnini AL, Machado MA. Carcinoid tumor of the gallbladder. Sao Paulo Med J. 1998 May-Jun;116(3):1741-3. doi: 10.1590/s1516-31801998000300010. PMID: 9876454.
43. Christian N. Meyer, Jan Geerdsen, Nils Christensen; Limited Surgical Excision of a Localized Carcinoid Tumour of the Cystic Duct. Dig Surg 1 May 1997; 14 (5): 423–425. https://doi.org/10.1159/000172587
44. Mochizuki M. Minute carcinoid tumor of the gallbladder. Acta Pathol Jpn. 1991 May;41(5):383-5. doi: 10.1111/j.1440-1827.1991.tb01662.x. PMID: 1678242.
45. Moraes AB, Treistman N, Studart MC, Chagas VLA, Brabo EP, Vieira Neto L. Gastrinoma of Cystic Duct: A Rare Association With Multiple Endocrine Neoplasia Type 1. J Clin Med Res.

2018 Nov;10(11):843-847. doi: 10.14740/jocmr3541w. Epub 2018 Oct 9. PMID: 30344820; PMCID: PMC6188020.

46. Morelli L, Piscioli F, Cudazzo E, Del Nonno F, Licci S. Simultaneous occurrence of metastasizing carcinoid tumour of the gallbladder and chromophobe renal cell carcinoma in a young man. Acta Gastroenterol Belg. 2007 Oct-Dec;70(4):371-3. PMID: 18330096.
47. Naito S, Naito M, Yamamoto N, Kume T, Hosino S, Kinjyo Y, Naito Y, Naito H, Hasegawa S. Polypoid gallbladder neuroendocrine tumor diagnosed as benign polyp before surgery: A case report. Mol Clin Oncol. 2020 Mar;12(3):225-229. doi: 10.3892/mco.2019.1971. Epub 2019 Dec 24. PMID: 32064098; PMCID: PMC7017218.
48. Naseer F, Kabir M. Carcinoid tumour of gall bladder. J Pak Med Assoc. 1992 Sep;42(9):227-8. PMID: 1433813.
49. Nicolescu PG, Popescu A. Carcinoid tumor of the cystic duct. Morphol Embryol (Bucur). 1986 Oct-Dec;32(4):275-7. PMID: 2949144.
50. Nishigami T, Yamada M, Nakasho K, Yamamura M, Satomi M, Uematsu K, Ri G, Mizuta T, Fukumoto H. Carcinoid tumor of the gall bladder. Intern Med. 1996 Dec;35(12):953-6. doi: 10.2169/internalmedicine.35.953. PMID: 9030993.
51. Ozawa K, Kinoshita M, Kagata Y, Matsubara O. A case of double carcinoid tumors of the gallbladder. Dig Dis Sci. 2003 Sep;48(9):1760-1. doi: 10.1023/a:1025499112957. PMID: 14560997.
52. Porter JM, Kalloo AN, Abernathy EC, Yeo CJ. Carcinoid tumor of the gallbladder: laparoscopic resection and review of the literature. Surgery. 1992 Jul;112(1):100-5. PMID: 1535733.
53. Raptis D, Savvides E, Langas G, Chatzimavroudis G, Papaziogas B. Neuroendocrine neoplasm of the cystic duct: report of two cases and literature review. Hippokratia. 2021 Jul-Sep;25(3):141-144. PMID: 36683907; PMCID: PMC9851141.
54. Resnick MB, Jacobs DO, Brodsky GL. Multifocal adenocarcinoma in situ with underlying carcinoid tumor of the gallbladder. Arch Pathol Lab Med. 1994 Sep;118(9):933-4. PMID: 8080366.
55. Saxton CR. Unusual presentation of carcinoid tumor as acute cholecystitis. South Med J. 1983 Jul;76(7):947-8. doi: 10.1097/00007611-198307000-00041. PMID: 6867811.
56. Shah IA, Schlageter MO, Wong SX, Gani OS. Carcinoid tumor of the cystic duct. Dig Surg. 1998;15(4):372-3. doi: 10.1159/000018635. PMID: 9845617.
57. SHIFFMAN M, JULER G. CARCINOID OF THE BILIARY TRACT. Arch Surg. 1964 Dec;89:1113-5. doi: 10.1001/archsurg.1964.01320060181033. PMID: 14208462.
58. Sinkre PA, Murakata L, Rabin L, Hoang MP, Albores-Saavedra J. Clear cell carcinoid tumor of the gallbladder: another distinctive manifestation of von Hippel-Lindau disease. Am J Surg Pathol. 2001 Oct;25(10):1334-9. doi: 10.1097/00000478-200110000-00017. PMID: 11688471.
59. Solanki R, Singh H, Kumar R. Exceptional Visualization of the Gallbladder on 68Ga-DOTANOC PET/CT Imaging. Clin Nucl Med. 2022 Jun 1;47(6):e468-e469. doi: 10.1097/RLU.0000000000004157. Epub 2022 Mar 30. PMID: 35353720.
60. Stavridi F, Chong H, Chan S, Goldsmith C, Reddy M, Glees J, Benepal T. Neuroendocrine tumour of the cystic duct: a case report and literature review. J Gastrointest Cancer. 2007;38(1):32-3. doi: 10.1007/s12029-008-9013-3. PMID: 19065721.
61. Sumiya R, Shimizu A, Nagai T, Ono H, Kumazawa K, Endo D, Oide T, Aoyanagi N. Clear cell neuroendocrine tumor in the gallbladder diagnosed as a benign polyp preoperatively: a case report. World J Surg Oncol. 2021 Jan 2;19(1):3. doi: 10.1186/s12957-020-02104-2. PMID: 33388069; PMCID: PMC7778816.

62. Sánchez Chiroboya MC, Morón García BI, Brox Torrecilla N, Palomino Donayre HO, Miguélez González M. Ultrasound calcifications in gallbladder lesions as a sign of suspected neuroendocrine tumour of the gallbladder. Endocrinol Diabetes Nutr (Engl Ed). 2023 May;70(5):367-369. doi: 10.1016/j.endien.2023.05.005. Epub 2023 May 22. PMID: 37225621.
63. Tanaka K, Iida Y, Tsutsumi Y. Pancreatic polypeptide-immunoreactive gallbladder carcinoid tumor. Acta Pathol Jpn. 1992 Feb;42(2):115-8. doi: 10.1111/j.1440-1827.1992.tb03085.x. PMID: 1561882.
64. Tasci HI, Coskunoglu EZ, Turk E, Karagulle E. Neuroendocrine Tumour of the Gallbladder Diagnosed after Cholecystectomy. J Coll Physicians Surg Pak. 2022 Aug;32(8):S127-S129. doi: 10.29271/jcpsp.2022.Supp2.S127. PMID: 36210670.
65. Tomihara H, Hashimoto K, Wakasa T, Ishikawa H, Tsujimoto T, Gakuhara A, Fukuda S, Ohta K, Kitani K, Hida JI, Ohta Y, Yukawa M. Successful resection of a neuroendocrine tumor in the gallbladder: a case report. Surg Case Rep. 2020 Oct 27;6(1):274. doi: 10.1186/s40792-020-01055-w. PMID: 33108599; PMCID: PMC7591665.
66. Vanoli A, Messina A, Gallotti A, Fugazzola P, Adsay V. Well-Differentiated Neuroendocrine Tumor of the Gallbladder with Paraganglioma-Like Features: a Potential Mimicker of Gallbladder Paraganglioma. Endocr Pathol. 2023 Sep;34(3):358-360. doi: 10.1007/s12022-023-09784-y. Epub 2023 Aug 21. PMID: 37599339.
67. Wang P, Chen J, Jiang Y, Jia C, Pang J, Wang S, Chang X. Neuroendocrine Neoplasms of the Gallbladder: A Clinicopathological Analysis of 13 Patients and a Review of the Literature. Gastroenterol Res Pract. 2021 May 22;2021:5592525. doi: 10.1155/2021/5592525. PMID: 34122537; PMCID: PMC8166508.
68. Yokoyama Y, Fujioka S, Kato K, Tomono H, Yoshida K, Nimura Y. Primary carcinoid tumor of the gallbladder: resection of a case metastasizing to the liver and analysis of outcomes. Hepatogastroenterology. 2000 Jan-Feb;47(31):135-9. PMID: 10690596.
69. Yoshikawa, Yasuji & Masuda, Takashi. (2020). A case of lipid-rich neuroendocrine tumor of the gallbladder mimicking a cholesterol polyp. Human Pathology: Case Reports. 20. 200373. 10.1016/j.ehpc.2020.200373.
70. Zou YP, Li WM, Liu HR, Li N. Primary carcinoid tumor of the gallbladder: a case report and brief review of the literature. World J Surg Oncol. 2010 Feb 23;8:12. doi: 10.1186/1477-7819-8-12. PMID: 20175936; PMCID: PMC2834672.
