# Supplementary material for: Neuroendocrine Tumors of the Gallbladder: A Multicenter Case Series and Systematic Literature Review Indicating Predominantly Non-Aggressive Tumor Behavior and a Common Association with Cholesterol Polyps and Cholesterolosis
Source: Endocr Pathol. 2026 Jun 11;37(1):26. doi: 10.1007/s12022-026-09921-3 (PMC13260158; doi:10.1007/s12022-026-09921-3)
Supplement: Supplementary file 6 — Supplementary Material 5 (PDF 512 KB) [file 12022_2026_9921_MOESM5_ESM.pdf]

	Cystic duct/gallbladder neck NETs (n=13)	Body/fundus NETs (n=12)	P-value
**Male sex, n (%)**	1 (8%)	8 (67%)	**0.004**
**Median patient age (range)**	50 years (23-88)	50 years (27-75)	0.904
**Hereditary tumor syndrome, n (%)**	0	1 (MEN1)	0.480
**Gross pattern**			**0.005**
Polypoid growth, n (%)	3 (23)	10 (83)	
Nodular/mural growth, n (%)	10 (77)	2 (17)	
**Tumor size, median (range)**	0.6 cm (0.08-1,9)	0.8 cm (0.4-2.3)	0.280
**WHO tumor grade, n (%)**			0.645
G1, n (%)	11 (85)	9 (75)	
G2, n (%)	2 (15)	3 (25)	
**pT stage***			0.111
pT1, n (%)	3 (23)	7 (58)	
pT2, n (%)	10 (77)	5 (42)	
**pN stage***			1.0
pN0, n (%)	6 (100)	2 (100)	
pN1, n (%)	0	0	
**Other gallbladder pathologies**			
Cholelithiasis, n (%)	7 (54)	3 (23)	0.226
Cholesterolosis, n (%)	3 (23)	6 (50)	0.226
Adenomyoma, n (%)	0	1 (8)	0.480
Gastric and/or intestinal metaplasia, n (%)	3 (23)	5 (42)	0.411
Low-grade dysplasia, n (%)	2 (15)	0	0.480
ICPN with associated adenocarcinoma, n (%)	0	1 (8)	0.480
Hyperplasia-like neuroendocrine proliferations, n (%)	1 (8)	0	1.0
Legend: ICPN: intracholecystic papillary-tubular neoplasm; MEN1 (multiple endocrine neoplasia type 1). *according the UICC staging system (9th ed.) of gallbladder carcinoma. No lymph node submitted in 6 cystic duct/gallbladder neck and in 9 fundus/body cases.			

Supplementary Table 1. Clinicopathologic Features of Gallbladder Neuroendocrine Tumors (GB-NETs) from the Multicenter Cohort, Stratified by Tumor Site
